# Breast Cancer Surgery During the COVID-19 Pandemic Peak in the UK: Operative Outcomes

**DOI:** 10.7759/cureus.9280

**Published:** 2020-07-19

**Authors:** Emma G MacInnes, Jenny Piper, Catherine Tait, Alison Waterworth, Raj Achuthan, Brian Hogan, Shireen McKenzie, Philip Turton, Baek Kim, Kieran Horgan

**Affiliations:** 1 Breast Surgery, Leeds Teaching Hospitals NHS Trust, Leeds, GBR; 2 Breast Surgery, York Teaching Hospitals NHS Foundation Trust, York, GBR; 3 Breast Surgery, Bradford Teaching Hospitals NHS Foundation Trust, Bradford, GBR; 4 Breast Surgery, Calderdale and Huddersfield NHS Foundation Trust, Huddersfield, GBR

**Keywords:** breast cancer, covid-19, breast surgery

## Abstract

Introduction

The COVID-19 pandemic caused widespread changes in delivery of breast cancer care, aiming to protect vulnerable patients whilst minimising compromise to oncological outcomes. This multicentre observational study aimed to establish early surgical outcomes from breast cancer surgery performed during the peak of the COVID-19 pandemic.

Materials and methods

Data were collected on consecutive patients that underwent breast surgery in four units between 16 March and 24 April 2020. Outcome data at 30 days post-operation were collected, including documented COVID-19 cases in patients and reported cases in healthcare workers directly involved in their care. Recommended modifications to practice to reduce COVID-19 transmission risk, both to patients and healthcare workers in each centre, are described.

Results

A total of 202 patients underwent surgery in four hospitals delivering breast services in the West Yorkshire region over the six-week period at the peak of the pandemic. The age ranged from 28 to 91 years (median 57, interquartile range, 48-65) with 22% having co-morbidities linked to COVID-19, e.g. diabetes or respiratory disease. No patients presented post-operatively with COVID-19 symptoms and at 30 days there had not been any identified COVID-19 cases. There were no unexpected critical care admissions or deaths. One healthcare worker involved in the delivery of breast surgery was diagnosed with COVID-19 during this time and made an uneventful recovery.

Conclusion

Breast cancer surgery, in selected groups and with meticulous adherence to measures designed to reduce COVID-19 transmission, does not appear to be associated with elevated risk to patients or healthcare workers.

## Introduction

Breast cancer is the most common cancer in women in the UK with 80% of patients undergoing surgery [1]. The incidence rises with age, and therefore many patients may have other associated medical comorbidities. Cancer treatment has been reported as being an independent risk factor both for COVID-19 infection and for a clinically severe disease course and death [2-6]. Some of these conclusions are based on studies with small patient numbers and significant heterogeneity of diagnosis and procedure and raised uncertainty about how this association should be extrapolated to specific cancers [7,8]. Increasing age, co-morbidities, particularly cardiorespiratory disease, diabetes and obesity, surgery and chemotherapy are additionally associated with an increase in COVID-19 risks, with particular emphasis on both age and co-morbidities in more recently published work [2,3,6,9-11].

When surgery is undertaken in the COVID-19 situation, it must offer effective oncological management with the lowest infection exposure risks, aiming to minimise patients’ length of stay and requirement for post-operative visits [12]. Procedures such as immediate breast reconstruction were suspended, and many units stopped offering complex breast remodelling procedures, such as therapeutic mammoplasties, where operative time is increased and in particular wound healing complications are more frequently encountered which may necessitate hospital visits and further surgery [13]. Additionally, staffing levels, availability of theatre and anaesthetic equipment needed to be balanced carefully against the need to deliver emergency care.

Service delivery has changed rapidly to minimise risk to both patients and healthcare workers. Some units moved from a COVID-receiving 'hot' site to an elective-only facility, whilst others had ring-fenced elective beds and theatres in a 'green area' (or 'cold area') within a COVID-receiving hospital. Extended use of personal protective equipment (PPE) in patient-facing environments and particularly in operating theatres has become the new norm with changes to outpatient clinics designed to reduce exposure and risk of transmission.

This study describes early experience of breast cancer surgery patient outcomes in terms of morbidity and mortality during the COVID-19 situation in four different hospitals.

## Materials and methods

Four breast units in West Yorkshire report consecutive patients undergoing breast cancer surgery undertaken over a six-week period between 16 March and 24 April 2020, during the peak of the pandemic in the region [14]. Data were collected from prospectively maintained hospital electronic records across all four NHS trusts. Data include patient details (age at surgery, co-morbidities: pre-existing respiratory and cardiovascular disease, diabetes or immunosuppression of any aetiology; BMI; menopausal status), surgery details (procedure; type of anaesthetic; length of stay; post-operative complications including returns to theatre; re-admission; unplanned critical care admission; COVID-19 infection and death) and pre-operative summary of tumour biology (imaging size and where appropriate, biopsy grade and receptor status). Data on post-operative COVID-19 status were based upon a lack of reported symptoms by patients and the absence of a positive result on any diagnostic testing (PCR, antibodies or imaging with findings typical of COVID-19). No routine COVID-19 tests were performed in the post-operative period in any of the four units. Descriptive statistical analyses have been performed. Data on healthcare worker COVID-19 infections were taken from each unit based upon reported sick leave.

All four units had strategies for continuing breast cancer assessment and treatment during the COVID-19 pandemic that were regularly revised, guided by local management to accommodate COVID-19 admissions, workforce reallocation and elective theatre capacity. Additionally, recommendations from the Association of Breast Surgery, The Royal Colleges of Surgeons and NHS England regarding use of PPE, case prioritisation and pre-operative COVID-19 screening were adopted [12,15,16]. This study pre-dated guidance requiring patients to self-isolate for two weeks prior to surgery, but patients were encouraged to isolate by the clinical teams in all four units.

Peri-operative adaptations

Theatre capacity was reduced in all four units to allow for staffing re-allocation and in preparation for the anticipated pandemic-peak impact on hospital services. Full PPE was worn by all operating surgeons and assistants, anaesthetists and scrub team, including fit-tested filtering face piece respirator type masks (in three of four Trusts), disposable fluid-resistant gowns and eye/face protection. Operating lists were adjusted to reduce the number of patients on a list to allow more time between cases for patient movement and observing social distancing. During intubation and extubation, any personnel not required to be in the room were asked to leave, with a period of time to allow air recirculation before access was permitted. Patients were discharged on the day of surgery wherever possible.

## Results

**Figure 1:**
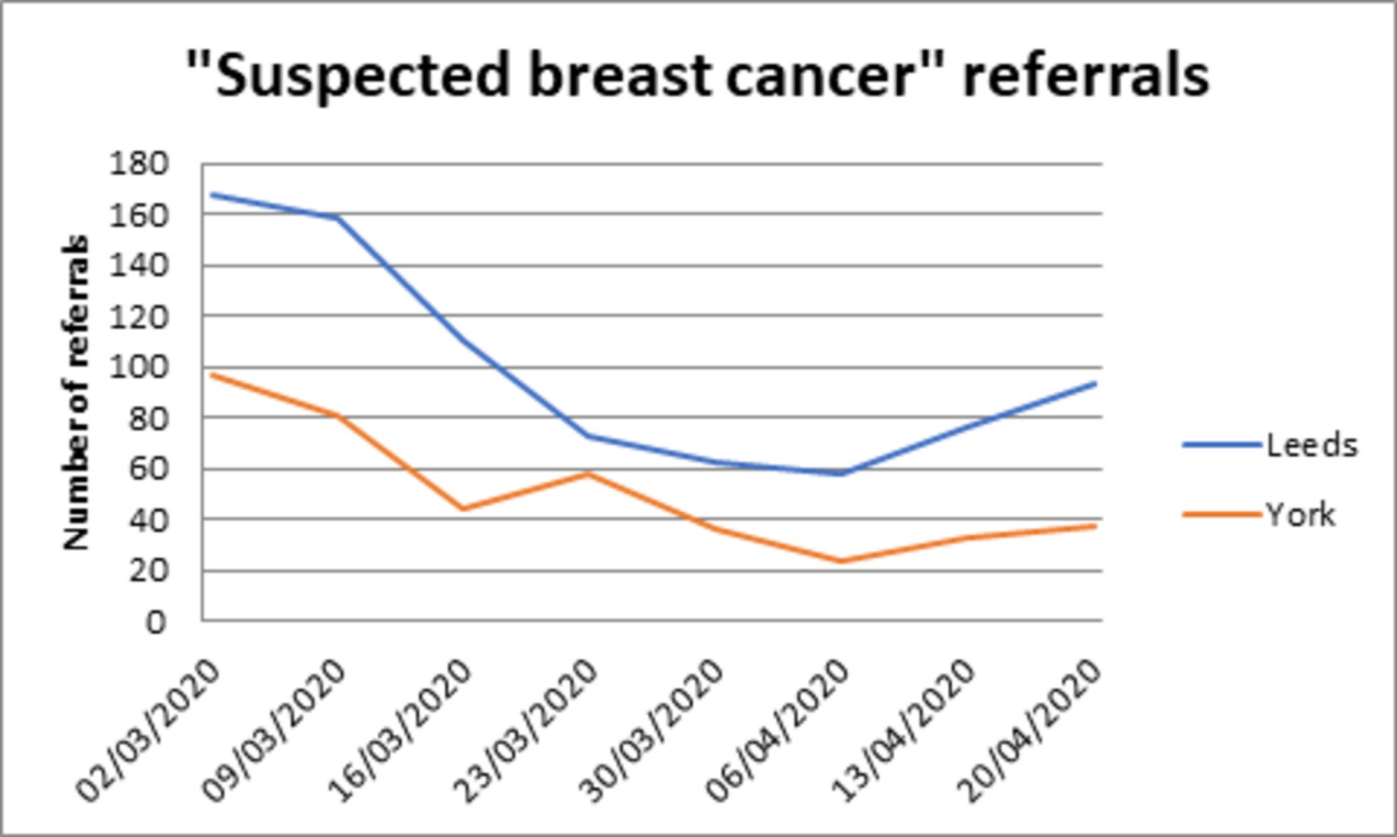
Referral pattern to Leeds and York Breast units over the peak of the pandemic

**Figure 2:**
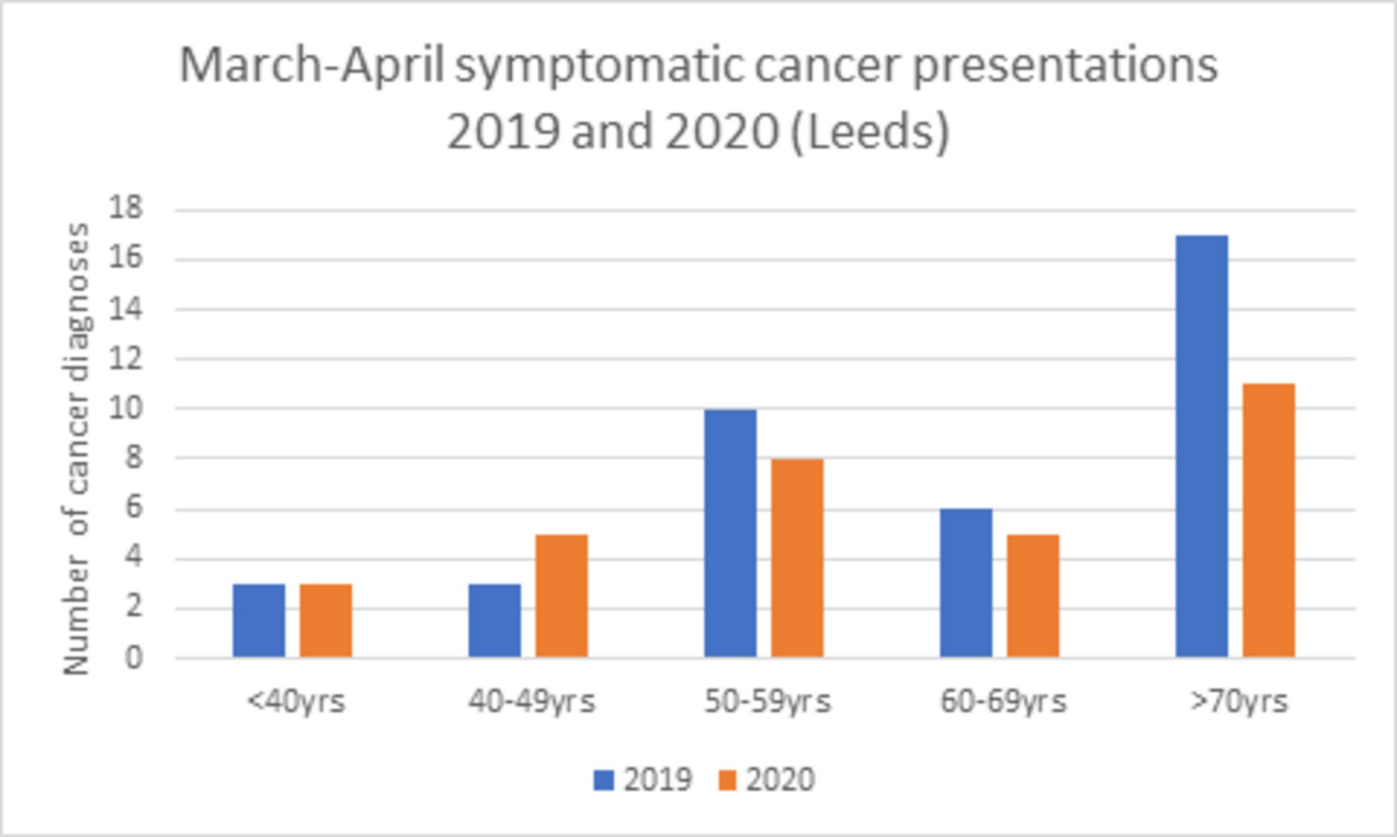
Cancer diagnoses in Leeds 2019 and 2020

Clinic referral rates decreased during the study period to approximately one-third (Figure 1). The reduction in new cancer diagnoses was, however, small (32 symptomatic cancers diagnosed in Leeds in a six-week period including the pandemic peak compared with 39 across the same time period in 2019). The number of patients diagnosed with breast cancer was comparable across all age groups, apart from those over 70 years of age (Figure 2). Although NHS breast screening did stop during the study period, recent screening diagnoses continued to be seen and treated from screening undertaken prior to the COVID-19 restrictions. Additionally, during the study period, 27 patients underwent surgery who had been receiving neoadjuvant chemotherapy and had either completed this treatment or in whom proceeding to earlier surgery was felt more appropriate due to concern about the immunosuppressing effects of chemotherapy and the lack of an apparent response. 

Across all four sites, a total of 202 operations were undertaken between 16 March and 24 April 2020 (Table 1). Pre-operative COVID screening rate was variable in the four breast units (Table 2), with some adopting routine screening after the study period. Patients' age ranged from 28 to 91 years, and 200/202 (99%) procedures required general anaesthetic and 146/202 (72%) were discharged on the day of surgery. 44/202 (22%) had a comorbidity associated with increased COVID-19 risk. BMI ranged from 17.5 to 55.2 with a median of 29.1 (Table 3). There were no unexpected post-operative critical care admissions or readmissions, no evidence of post-operative COVID-19 infection and no deaths. There was one single reported COVID case in a healthcare worker directly involved in patient care over this period who made an uneventful recovery without requiring hospitalisation. A total of 56 patients were given ‘bridging’ endocrine therapy across the four sites, that is, with a plan for surgery once concern about COVID-19 transmission was reduced. 

**Table 1:** Breast unit details

	Bradford	Huddersfield and Calderdale	Leeds	York
Theatres within a ‘cold’ part of a COVID-receiving hospital	11	55	75	10
Moved practice to a non-COVID-receiving hospital (private healthcare provider)	16	0	0	35
All breast operations same time period 2019	59	84	124	63

**Table 2:** Pre-operative screening practice

	Bradford	Huddersfield and Calderdale	Leeds	York
Nasopharyngeal swab	Screening commenced after the study period	Screening commenced after the study period. One positive result in a symptomatic patient undergoing neoadjuvant chemotherapy – surgery postponed	Screening commenced on 02/04/2020. 35/84 patients tested one positive result – surgery postponed	Screening commenced on 04/04/2020. 3/45 patients tested. No positive results
Chest x-ray	Not used	Not used	Screening commenced on 08/04/2020. 29/84 performed. All reported as no evidence of COVID-19	Not used

**Table 3:** Surgery details 16 March 2020 until 24 April 2020 *Average median value. N/S = not stated. Body mass index, which is routinely calculated in pre-assessment, was not always measured as more consultations were conducted by telephone.

		Bradford (n=27 patients)	Huddersfield and Calderdale (n=51 patients)	Leeds (n=74 patients)	York (n=45 patients)	Total (n=202 patients)
Total number of breast operations		27	55	75	45	202
Age	Range (IQR)	30-91 (46-64)	34-85 (47-65)	28-82 (45-62)	41-86 (56-68)	28-91 (48-65)
	Median	57	56	55	61	57*
Anaesthetic	General	26	55	74	45	200
	Local	1	0	1	0	2
Respiratory/cardiovascular co-morbidities, diabetes or immunosuppression		13 (48%)	4 (8%)	24 (32%)	3 (7%)	44 (22%)
Body mass index	Mean	30.1	N/S	28.2	29.0	29.1
	Range	21-48	N/S	17.5-55.2	19.7-42	17.5-55.2
Length of stay	Day case	19 (70%)	34 (62%)	53 (71%)	40 (89%)	146 (72%)

A total of 171 breast cancer resection surgeries were undertaken, 66 (39%) were simple mastectomy and 105 (61%) breast conservation of which 10 were therapeutic mammoplasties. A total of 126 women underwent sentinel lymph node biopsy with 44 axillary node clearances, three of whom had returned for completion axillary surgery during the study period. Two patients required a return to theatre to manage post-operative haematoma (<1%), and there were no unplanned critical care admissions and no recorded symptoms suggestive of COVID or PCR diagnoses in the post-operative period. Table 4 highlights the tumour characteristics of the patient cohort. 

**Table 4:** Invasive tumour characteristic for patients who underwent surgery (excluding 16 patients with ductal carcinoma in situ)

		Bradford (n=24)	Huddersfield and Calderdale (n=52)	Leeds (n=67)	York (n=43)	Total (n=186)
Grade	1	1	6	6	3	16 (9%)
	2	12	34	37	16	99 (53%)
	3	11	11	22	22	66 (35%)
	Not stated	0	1	2	2	5 (3%)
Oestrogen receptor positive		17 (71%)	39 (75%)	52 (78%)	29 (67%)	137 (74%)
HER2 positive		3 (13%)	11 (21%)	19 (28%)	7 (16%)	40 (22%)
Triple receptor negative		7 (29%)	10 (19%)	9 (13%)	10 (23%)	36 (19%)
Post-menopausal, oestrogen receptor positive		13 (54%)	30 (58%)	34 (51%)	25 (58%)	102 (55%)
Post neoadjuvant chemotherapy		6	5	11	5	27 (14%)
Maximum size on pre-operative imaging	<2 cm	9	25	29	14	77 (41%)
	>2 cm	15	27	36	22	100 (54%)
	Not stated	0	0	2	7	9 (5%)

## Discussion

Previous studies documented the elevated risk of COVID-19 in the peri-operative period with relatively poorer outcomes for patients undergoing surgery [2,3,6,9]. By combining major and minor procedures, different modes of anaesthesia, elective and emergency operating, and outcomes from all surgical specialties, inherent heterogeneity limits the extent to which findings can be used to guide practice in an individual speciality and this is particularly the case for peri-operative COVID-19 risks [6]. These were rapid reports, designed to inform in a fast-changing landscape in which clinicians and patients needed guidance. With the benefit of this information, greater care has been taken to minimise risk and we report good outcomes for this series of 202 patients, with no identified post-operative COVID-19 related symptoms or infections and no deaths associated with surgery. Cancer patients were previously identified as being at greater risk, but this risk elevation was not apparent in this series [2-6]. Of note, 102 patients were post-menopausal with oestrogen receptor positive disease who could have been managed with neoadjuvant endocrine therapies but who chose to proceed with surgery after careful consideration of risks and benefits. Only 9% of patients had grade 1 cancer, whilst 35% were grade 3, suggesting a selection bias for proceeding with surgery for higher risk cancers in keeping with guidelines.

Only one of the breast units reported any breast surgery department healthcare worker confirmed COVID-19 infection and in this single case the doctor (an anaesthetist) isolated and made an uneventful recovery. The denominator for this one case is hard to establish with staff moving between roles during this time. The nature of this type of data is inherently weak; however, the fact that none of the four units had more than this one case is reassuring.

In total, 38 patients had pre-operative screening for COVID-19. There were two positive pre-operative results, one in a patient undergoing neoadjuvant chemotherapy (symptomatic) and one in an asymptomatic patient screened pre-operatively. Both had negative repeat tests and surgery went ahead without complications and with no post-operative morbidity or mortality from COVID-19.

In all four units, women with hormone-sensitive disease were offered the option of delaying surgery with use of oral anti-oestrogen medication balanced against their individual risks of proceeding with surgery, taking into account both patient and tumour factors. The lack of locally or nationally reported previous figures for neoadjuvant endocrine therapy means it is hard to compare, but it is felt that a significantly higher number of women started this treatment than would in a non-COVID period.

When the referral data were examined across the units, the reduction in new diagnoses appears to be mainly in those over the age of 70 years, who were strongly advised to self-isolate in the UK. The number of operations performed over the same time period in 2019 demonstrates a 38% reduction. The majority of procedures accounting for this difference were elective, revisional and reconstructive, with the proportion of cancer patients proceeding to surgery remaining relatively stable. Although 56 patients started bridging endocrine treatment, there are also patients who would ordinarily have been offered to start or continue neoadjuvant chemotherapy and who were, in line with guidance, instead advised to proceed to surgery, likely accounting for this relative stability.

There are ethical implications to offering surgery during a life-threatening viral pandemic of this nature. Use of theatre, anaesthetic, PPE and valuable staffing resources need to be considered and reviewed regularly. The favourable outcomes in this series have been collected and analysed prospectively, and every care taken to minimise risk. Interdepartmental communication between clinicians and managers has been crucial to ensuring safe delivery of care. As we move into a COVID-19 ‘recovery’ period, with increasing resumption of elective services, the outcomes from this study may be used to support the delivery of more minor and intermediate risk procedures that had been discontinued, with obvious implications for workload and surgical planning.

Limitations include the fact that post-operative COVID-19 diagnoses and complications have been recorded from electronic case records in the participating trusts but do not exclude the possibility of a patient having a positive result at a different hospital. With regular contact from the breast care nursing team, however, it seems unlikely that a significant event would have gone unrecorded. The variance in reported co-morbidities may reflect a methodological weakness associated with electronic case-note review. A strength of this study is the inclusion of patients receiving care in units with different risk management strategies and a variety of ‘hot’ and ‘cold’ facilities but with consistently good outcomes. There are inherent limitations to any observational study, with selection bias based upon individual risk management decisions of patients and clinicians; however, the findings do offer reassurance that the risks associated with breast surgery during the COVID-19 pandemic do not appear to be as high as were reported in previous series of cancer surgery outcomes.

## Conclusions

Despite the pressures associated with the COVID-19 pandemic, breast cancer surgery can be safely delivered. Combined with a stringent protocol to reduce COVID-19 transmission and exposure, breast cancer surgery does not appear to be associated with an elevated risk of post-operative morbidity or mortality. It is important that healthcare workers and patients meticulously observe measures designed to reduce COVID-19 transmission. Clinicians must discuss with patients the known risks of attending hospital for assessment and treatment and the additional concern regarding elevated risks of a poor outcome if COVID-19 is diagnosed in a post-operative period in a patient with a cancer diagnosis. However, patients can be reassured that risk mitigation approaches can successfully reduce the risk of an adverse outcome.
